# A pilot study of a deep learning approach to detect marginal bone loss around implants

**DOI:** 10.1186/s12903-021-02035-8

**Published:** 2022-01-16

**Authors:** Min Liu, Shimin Wang, Hu Chen, Yunsong Liu

**Affiliations:** Department of Prosthodontics, Peking University School and Hospital of Stomatology and National Engineering Laboratory for Digital and Material Technology of Stomatology and Research Center of Engineering and Technology for Digital Dentistry of Ministry of Health and Beijing Key Laboratory of Digital Stomatology and National Clinical Research Center for Oral Diseases, 22 ZhongguancunNandajie, Haidian District, Beijing, 100081 China

**Keywords:** Artificial intelligence, Deep learning, Dental implant, Marginal bone loss

## Abstract

**Background:**

Recently, there has been considerable innovation in artificial intelligence (AI) for healthcare. Convolutional neural networks (CNNs) show excellent object detection and classification performance. This study assessed the accuracy of an artificial intelligence (AI) application for the detection of marginal bone loss on periapical radiographs.

**Methods:**

A Faster region-based convolutional neural network (R-CNN) was trained. Overall, 1670 periapical radiographic images were divided into training (n = 1370), validation (n = 150), and test (n = 150) datasets. The system was evaluated in terms of sensitivity, specificity, the mistake diagnostic rate, the omission diagnostic rate, and the positive predictive value. Kappa (κ) statistics were compared between the system and dental clinicians.

**Results:**

Evaluation metrics of AI system is equal to resident dentist. The agreement between the AI system and expert is moderate to substantial (κ = 0.547 and 0.568 for bone loss sites and bone loss implants, respectively) for detecting marginal bone loss around dental implants.

**Conclusions:**

This AI system based on Faster R-CNN analysis of periapical radiographs is a highly promising auxiliary diagnostic tool for peri-implant bone loss detection.

## Introduction

Dental implants are important for restoring biological function in patients with missing teeth [1, 2] and have become increasingly popular since the 1980s [3]. Monitoring and maintenance are critical for long-term stability after implantation [4]. Marginal bone resorption is an important parameter that should be monitored. Bone loss of < 1.5 mm at 1-year post-loading is generally considered acceptable, followed by the loss of 0.2 mm annually thereafter [5, 6]. In cases where bone loss exceeds this amount, careful investigation is needed, including in cases showing gradual loss after osseointegration. Bone loss is initiated and maintained by iatrogenic factors or local conditions (e.g. occlusal trauma, implant factors, prosthetic restorations, etc.) [5, 7, 8]. Bone loss can be classified into late and additional types [9]. By monitoring marginal bone resorption, early changes in clinical factors can be identified. When additional bone loss is observed along with peri-implant connective tissue inflammation (i.e. bleeding and/or suppuration), a diagnosis of peri-implantitis is made [10]. This requires treatment and oral health education for the patient.

Bone loss is usually evaluated on radiographs. A difference in measurements between examiners of approximately 1–2 mm is considered to reflect meaningful interexaminer variation [11]. For general practitioners, evaluating marginal bone loss around implants can be difficult. In clinical practice, detection of the peri-implant bone level relies on imaging findings. Commonly used imaging modalities include cone-beam computed tomography, panoramic radiography, and periapical radiography. Cone-beam computed tomography can depict the three-dimensional relationship between a dental implant and the surrounding alveolar bone, and studies have demonstrated robust accuracy of this modality for the detection of peri-implant bone defects [12, 13]. Other studies have sought to identify the bone condition around implants using periapical radiographs [14, 15]. Two-dimensional radiographic images are widely used in clinical practice because of their low cost and radiation dose; thus, bone defects are commonly measured on conventional periapical radiographs. Assessment of the peri-implant marginal bone level on conventional periapical radiographs is generally difficult because the three-dimensional bone shape is represented on a two-dimensional image. Therefore, the boundaries of the bone around the implant, as well as the buccal and lingual bone heights, should be determined by experienced clinicians [16]. Inexperienced clinicians may make diagnostic errors and false diagnoses according to clinical studies on learning curve [17]. Implant restoration is an increasingly popular procedure, but follow-up thereof can involve a considerable amount of clinical time and effort. Furthermore, interpretations of radiographs tend to vary among observers. Automated systems for reading and analysing periapical radiographs of dental implants may help to address these issues.

Recently, there has been considerable innovation in artificial intelligence (AI) for healthcare, which can also aid digital dentistry and telemedicine [18]. Convolutional neural networks (CNNs) show excellent object detection and classification performance [19]. Many studies based on CNNs have been conducted in the field of dentistry [20, 21], for tooth numbering [22] and analysis of dental caries [23], osteoporosis [24], periodontal bone loss [25], submerged primary teeth [26] and dental implants [27–29]. CNNs learn directly from raw input data and classify images without the requirement for manual feature extraction. Region-based convolutional neural networks (R-CNNs) have been developed for object detection tasks, whereby target objects (regions of interest) are automatically identified and annotated [30–33]. Subsequently, the R-CNN was upgraded to Faster R-CNN, which is more efficient. Based on Faster R-CNN, the Mask R-CNN method was developed; this can detect targets in images and provides high-quality segmentation results [34]. To our knowledge, few studies have used Faster R-CNN for detection of marginal bone loss around dental implants on periapical radiographs [28].

The purpose of this study was to develop an automated system for identifying marginal bone loss around dental implants in periapical radiographs using a deep learning-based object detection method, and then to investigate the accuracy of the system.

## Materials and methods

### Data collection and annotation

This study was approved by the bioethics committee of Peking University School and Hospital of Stomatology (PKUSSIRB-201837103). The study was conducted in accordance with institutional ethical guidelines. The data are anonymous, and the requirement for informed consent was therefore waived. In total, 2500 digital periapical radiographs of bone-level implants were collected from Peking University School and Hospital of Stomatology. The inclusion criteria were as follows: periapical radiographs of dental implants, appropriate radiation exposure, and radiographs of dental implants acquired in parallel. The exclusion criteria were as follows: excessively bright or dark images precluding distinguishment of marginal bone around dental implants, severely distorted images of dental implants, and/or graft material hindering observation of the alveolar bone [28]. Each digital radiograph was exported with a resolution of 96 dpi and size of approximately 300–500 × 300–400 pixels. Each radiograph was then rotated so that the implant was perpendicular to the horizontal plane and saved in JPG format image file with a unique identification code as a component of the primary dataset. All patient information (e.g. name, sex, and age) was removed from the images according to our previous experimental investigations [20, 22]. An experienced dentist (> 5 years of clinical experience) assessed the images for marginal bone loss around the dental implants. Overall, 835 images with marginal bone loss around the implants were detected and classified into the case group. The control group was then formed from 835 randomly selected radiographs from the primary dataset without marginal bone loss around the implants.

**Fig. 1:**
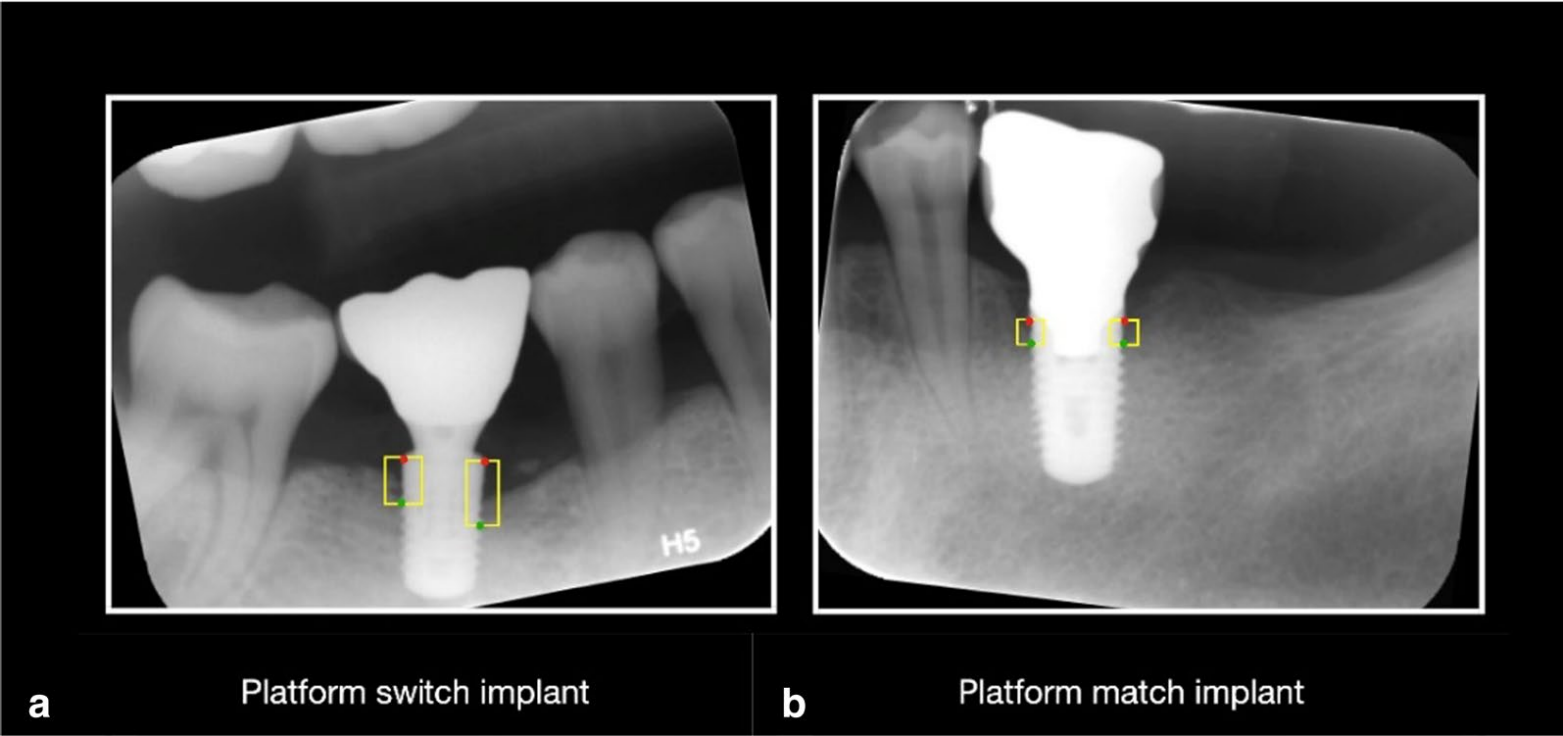
“Keypoints” for marginal bone loss assessment. **a** platform switch implant; **b** platform match implant. Red points indicate coronal keypoints; green points indicate apical keypoints. For platform-switched implants, the coronal keypoints were located on top of each implant. For bone-level platform-matched implants, the coronal keypoints were located on the bottom of the implant neck. The apical keypoints comprised the first point of contact between the bone and implant. The yellow bounding boxes denote areas of marginal bone loss

This study used a balanced dataset [26]. Images from the case and control group datasets were randomly assigned to one of three datasets: a training set of 1,370 images, a validation set of 150 images, and a test set of 150 images. The training and validation datasets were used to train a Faster R-CNN [32, 33]. Subsequently, the dentist with more than 5 years of clinical experience (reference standard) drew a rectangular bounding box around the dental implants and crowns, and around areas of marginal bone loss surrounding implants (ground truth bounding box for the case group). Another oral and maxillofacial radiologist confirmed the initial bounding box positions. During annotation, the clinicians drew the smallest possible bounding box around each area of marginal bone loss surrounding the implants in each image (Fig. 1).

For platform-matched implants, the bottom of the implant neck near the most coronal thread was considered as the top of the implant [7]. For platform-switched implants, the most coronal edge was considered as the top of the implant [14]. The apical “keypoints” were the first contact points of the bone and implant. Coordinates in the image were set in accordance with the distance from the top-left corner. The bounding box was described in terms of its top left and bottom right corners (xmin, ymin; xmax, ymax).

### Training and validation of the Faster R-CNN

An object detection package [33] for TensorFlow was used for object detection. Inception Resnet v2 (Atrous version), a state-of-the-art object detector, was used as the neural network model. The model was trained using a PC with a Quadro RTX 8000 graphics processing unit (NVIDIA, USA), 48 GB memory and 4608 CUDA cores. The backend algorithms were executed using TensorFlow (version 1.13.1) running on the Ubuntu 18.04 operating system.

A set of 1370 annotated X-ray images were used to train the Faster R-CNN for object recognition. There were 60,000 iterations and an initial learning rate of 0.0003, which was reduced to 0.00006 after 30,000 iterations.

To rapidly determine model performance, the average precision [35] (AP; i.e., the area under the curve) of the implant and marginal bone loss lesion areas, as well as the mean average precision (mAP) of an intersection over unit (IoU) of > 0.5, were calculated using the following equation:

$$ IOU = \frac{{Area_{pred} \cap Area_{gt} }}{{Area_{pred} \cup Area_{gt} }} $$

where Area_pred_ and Area_gt_ represent the predicted area of the bounding box and the ground truth bounding box, respectively. The IoU threshold was set at 0.5 because this value is commonly used in studies of object detection [36]. The mAP was calculated by determining the mean AP across all classes. Higher values indicated better learning system performance.

### Diagnostic performance analysis

The diagnostic accuracy of the model was determined by comparison with assessments performed by dentists. In total, 150 radiographic images were analysed by three dentists: a resident dentist (Dr1), an MD student with 2 years of experience (Dr2), and an experienced dentist (5 years of clinical experience; reference standard). Observers (Dr1 and Dr2) were asked to indicate areas of pathology and potential bone loss around implants on the images. The classification and detection performance of the AI system and observers was evaluated by comparison with the reference standard.

A confusion matrix (Table 1) summarising the predicted and actual results was used to determine the accuracy of the model. The sensitivity, specificity, mistake diagnostic rate, omission rate, and positive predictive value were calculated as follows:

$$ {\text{Sensitivity}}:S_{e} = \frac{a}{a + b} $$

$$ {\text{Specificity:}}S_{p} = \frac{d}{c + d} $$

$$ {\text{Mistake diagnostic rate}}:\hat{\alpha } = 1 - S_{p} = \frac{c}{c + d} $$

$$ {\text{Omission diagnostic rate}}:\hat{\beta } = 1 - S_{e} = \frac{b}{a + b} $$

$$ {\text{Positive predictive value}}:PV_{ + } = \frac{a}{a + c} $$

**Table 1:** Confusion matrix

Actual situation	Predicted situation	
	1	0
1	True-positive (a)	False-negative (b)
0	False-positive (c)	True-negative (d)

Interobserver agreement with respect to the presence/absence of marginal bone loss around implants was calculated using the kappa (κ) statistic in SPSS software (24; SPSS Inc., USA). The κ values were classified as follows: 0, poor; 0.00–0.20, weak; 0.21–0.40, fair; 0.41–0.60, moderate; 0.61–0.80, substantial; and 0.81–1.00, almost perfect agreement [37].

### Statistical analysis

The training and test datasets were used to create optimal weights for a deep CNN model. A confusion matrix was used to calculate the accuracy of the model, as stated above. The sensitivity, specificity, mistake diagnostic rate, omission diagnostic rate, and positive predictive value of the deep CNN model were calculated based on its performance with the test dataset, using a TensorFlow framework and Python. Interobserver agreement regarding the presence of marginal bone loss was given by the κ statistic, calculated in SPSS as also stated above.

## Results

**Fig. 2:**
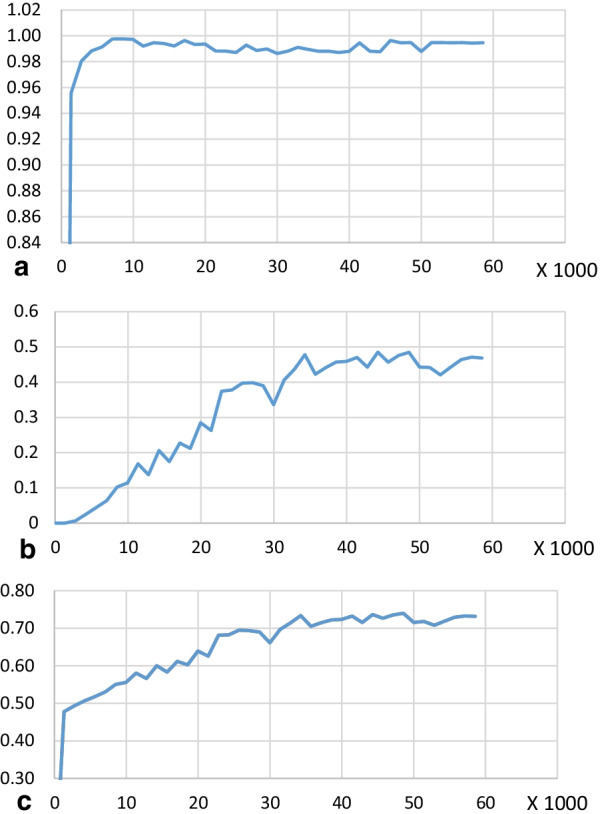
The average precision [35] (AP; i.e., the area under the curve) of the implant and marginal bone loss lesion areas, as well as the mean average precision (mAP) of an intersection over unit (IoU) of > 0.5, were calculated. **a** average precision of implant classification; **b** average precision of marginal bone loss lesion classification; **c** mean average precision

The AP for implants approached 0.99 after 10,000 iterations (Fig. 2a), indicating that the implants could be detected with high accuracy. The AP for marginal bone loss gradually increased with an increasing number of iterations. When the number of iterations reached 30,000, the AP value fluctuated slightly; it eventually stabilised at 0.47 after 60,000 iterations (Fig. 2b). The mAP of implants and marginal bone loss was 0.73 (Fig. 2c).

**Fig. 3:**
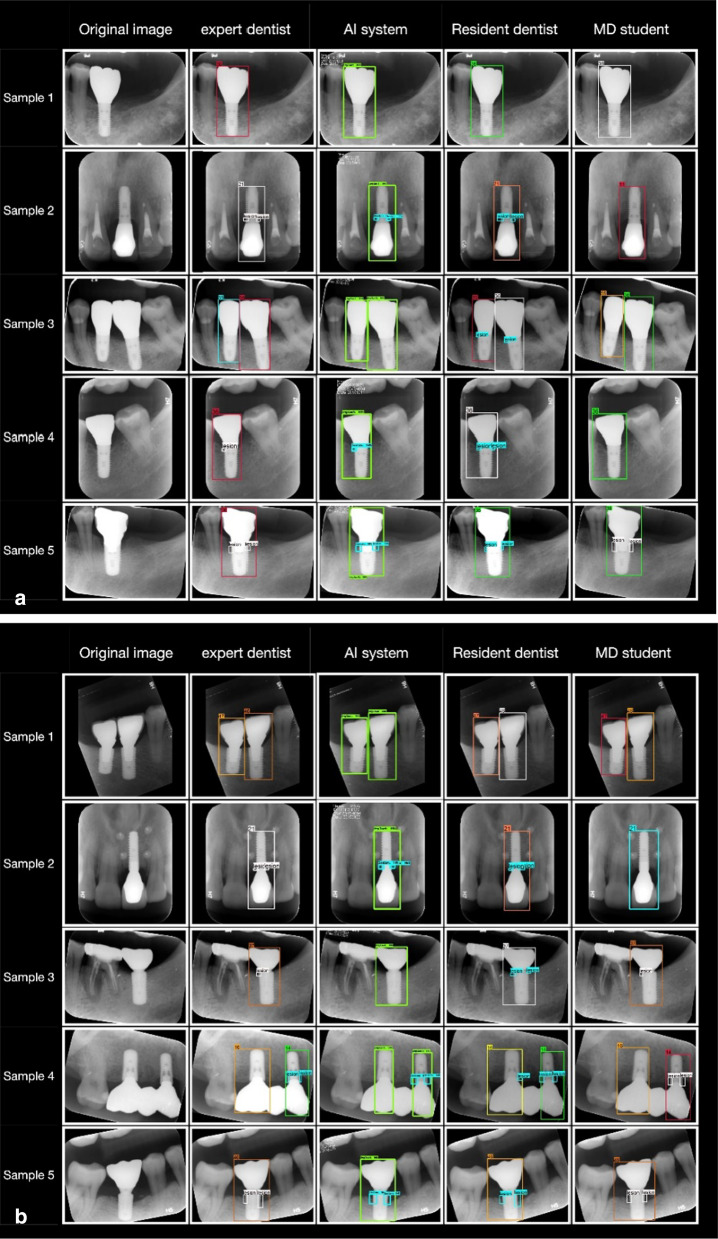
Example periapical radiographs showing areas of bone loss detected by neural networks. Images were manually annotated by an experienced dentist. **A** platform-matched implants. **B** platform-switched implants

Table 2 provides information on the implants in the training and test datasets. As shown in Fig. 3, although some diagnoses were missed, the bone loss area detected by Faster R-CNN was generally similar to the ground truth bounding box. With increasing severity of bone loss, the Faster R-CNN model and observer annotations converged.

**Table 2:** Implant classifications for the training and test datasets

implant-abutment connection type	Training data	Test data
Platform-switched	794	85
Platform-matched	875	111

Marginal bone resorption was assessed on the basis of single implants, as well as their mesial and distal sites. Table 3 compares the performance of the AI system and observers. For bone loss around implants and lesion sites, the deep CNN had positive predictive values of 81% and 87%, sensitivities of 67% and 75%, and specificities of 87% and 83%, respectively. The values for these parameters showed considerable variation between the observers.

**Table 3:** Performance comparison between the AI system and human observers

Metrics	Bone loss implants			Bone loss sites		
	AI (%)	Dr1 (%)	Dr2 (%)	AI (%)	Dr1 (%)	Dr2 (%)
Sensitivity	67	93	62	75	96	68
Specificity	87	64	77	83	55	72
Mistake diagnostic rate	13	36	23	17	45	28
Omission diagnostic rate	33	7	38	25	4	32
Positive predictive value	81	69	70	87	76	78

AI = artificial intelligence system; Dr1 = MD student; Dr2 = resident dentist

Notably, there was fair interobserver agreement (κ = 0.399 and 0.383 for bone loss sites and implants, respectively) between the MD student and expert dentist. However, the agreement between the AI system and expert was moderate to substantial (κ = 0.547 and 0.568 for bone loss sites and implants, respectively). Finally, there was moderate agreement (κ = 0.555 and 0.544 for bone loss sites and implants, respectively) between the resident dentist and expert dentist (Table 4).

**Table 4:** Interobserver agreement data

Comparison classification	System versus RS (κ)	Dr1 versus RS (κ)	Dr2 versus RS (κ)
Bone loss sites	0.547	0.555	0.399
Bone loss implants	0.568	0.544	0.383

Dr1 = MD student; Dr2 = resident dentist; RS = reference standard (experienced dentist)

## Discussion

AI technologies can be clinically evaluated in terms of diagnostic performance, patient outcomes, and the cost–benefit ratio [38, 39]. For many years, machine predictions were inferior to those of humans in terms of object detection and instance segmentation, and extensive comparisons of AI and human observers are lacking. In this study, implants were detected with high accuracy by the AI system. Marginal bone loss detection is often challenging, so several metrics of diagnostic performance were used for model evaluation in this study. Specificity represents the probability that a marginal bone loss bounding box actually contains the lesion area, while sensitivity represents the probability that an image is correctly labelled as “disease”. The κ statistic test is useful for evaluating consistency between a new diagnostic method and the gold standard; it can also be used to evaluate consistency between two clinicians in terms of their diagnostic assessments of specific patients. The above-described metrics allow for model evaluation and comparison among clinicians. The CNN model used in this study performed similarly to the resident dentist, but less well than the experienced dentist; however, overall we conclude that the CNN model may facilitate the detection of marginal bone loss around implants.

The impact of implant-supported prosthesis type on peri-implant bone loss and peri-implantitis remains unclear [7, 40]. The differential effects on loss of marginal bone between platform-matched and -switched implants has received increasing attention in recent years; a meta-analysis by Chrcanovic et al. [41] suggested that significantly less marginal bone loss occurs with the latter type of implant. Dentists must distinguish the abutment-implant connection type and appropriate reference points when analysing radiographs for marginal bone loss around dental implants. Platform-switched level implants should maintain marginal bone stability at a level equivalent to the top of the implant [14]. Platform-matched implants have a smooth neck, and the marginal bone should be stabilised at the junction between the smooth and rough implant surfaces [42]. In this study, we divided the marginal bone loss training data according to the implant-abutment connection type, and the bone resorption areas automatically identified by the CNN were generally consistent with these classifications (Fig. 3). These findings differed from those of Cha et al. [28], whose dataset included various implants with different implant-abutment junctions. In that study, the most coronal thread of the implant was used as a threshold position.

According to the VIII European Workshop on Periodontology [43], radiographs of implants are recommended after physiological remodelling (generally at the time of prosthesis fitting) to assess changes in the level of crestal bone. These baseline radiographs were unavailable for some patients in our dataset. Exposure of the rough implant surface can serve as an indicator of bone resorption around the implant. In this study, bounding boxes were used for qualitative detection of marginal bone loss (Fig. 2). The Faster R-CNN model was used in this study for feature detection and classification, while Cha et al. [28] used a Mask R-CNN model that detects and classifies targets by drawing target frames, and then segments targets at the pixel level. However, the cost of training is considerable because a set of keypoints must be precisely annotated for model training; also, specialised equipment is needed for training [34].

Although AI is a rapidly developing technology, our research nevertheless provides important baseline data for future studies. However, this study had some limitations. Firstly, for assessment of the real-world clinical performance of high-dimensional AI algorithms that analyse medical images using deep learning, external validation studies are needed [44–46]. This study used a balanced database, but the incidence of bone resorption at implant margins was low. Second, because subtle changes in marginal bone morphology are difficult to evaluate, standardised radiographs produced via the paralleling technique have important roles in monitoring marginal bone levels around endosseous implants [42]. Model performance may be improved by the parallel projection method.

## Conclusions

The Faster R-CNN model used in this study performed similarly to the resident dentist, but less well than the experienced dentist; overall we conclude that our Faster R-CNN could detect peri-implant bone loss on periapical radiographs and may facilitate the development of accurate diagnostic tools. In the future, model performance may be improved by more high qualified training images.

## Data Availability

The datasets used and analyzed during the current study are available from the corresponding author on reasonable request.
